# A study comparing the actions of gabapentin and pregabalin on the electrophysiological properties of cultured DRG neurones from neonatal rats

**DOI:** 10.1186/1471-2210-4-14

**Published:** 2004-08-04

**Authors:** David McClelland, Rhian M Evans, Louise Barkworth, Duncan J Martin, Roderick H Scott

**Affiliations:** 1 Department of Biomedical Sciences, Institute of Medical Sciences, The University of Aberdeen, Foresterhill, Aberdeen AB25 2RL, Scotland, UK

## Abstract

**Background:**

Gabapentin and pregabalin have wide-ranging therapeutic actions, and are structurally related to the inhibitory neurotransmitter GABA. Gabapentin, pregablin and GABA can all modulate voltage-activated Ca^2+ ^channels. In this study we have used whole cell patch clamp recording and fura-2 Ca^2+ ^imaging to characterise the actions of pregabalin on the electrophysiological properties of cultured dorsal root ganglion (DRG) neurones from neonatal rats. The aims of this study were to determine whether pregabalin and gabapentin had additive inhibitory effects on high voltage-activated Ca^2+ ^channels, evaluate whether the actions of pregabalin were dependent on GABA receptors and characterise the actions of pregabalin on voltage-activated potassium currents.

**Results:**

Pregabalin (25 nM – 2.5 μM) inhibited 20–30% of the high voltage-activated Ca^2+ ^current in cultured DRG neurones. The residual Ca^2+ ^current recorded in the presence of pregabalin was sensitive to the L-type Ca^2+ ^channel modulator, Bay K8644. Saturating concentrations of gabapentin failed to have additive effects when applied with pregabalin, indicating that these two compounds act on the same type(s) of voltage-activated Ca^2+ ^channels but the majority of Ca^2+ ^current was resistant to both drugs. The continual application of GABA, the GABA_B _receptor antagonist CGP52432, or intracellular photorelease of GTP-γ-S had no effect on pregabalin-induced inhibition of Ca^2+ ^currents. Although clear inhibition of Ca^2+ ^influx was produced by pregabalin in a population of small neurones, a significant population of larger neurones showed enhanced Ca^2+ ^influx in response to pregabalin. The enhanced Ca^2+ ^influx evoked by pregabalin was mimicked by partial block of K^+ ^conductances with tetraethylammonium.

Pregabalin produced biphasic effects on voltage-activated K^+ ^currents, the inhibitory effect of pregabalin was prevented with apamin. The delayed enhancement of K^+ ^currents was attenuated by pertussis toxin and by intracellular application of a (Rp)-analogue of cAMP.

**Conclusions:**

Pregabalin reduces excitatory properties of cultured DRG neurones by modulating voltage-activated Ca^2+ ^and K^+ ^channels. The pharmacological activity of pregabalin is similar but not identical to that of gabapentin. The actions of pregabalin may involve both extracellular and intracellular drug target sites and modulation of a variety of neuronal conductances, by direct interactions, and through intracellular signalling involving protein kinase A.

## Background

**Figure 1:**
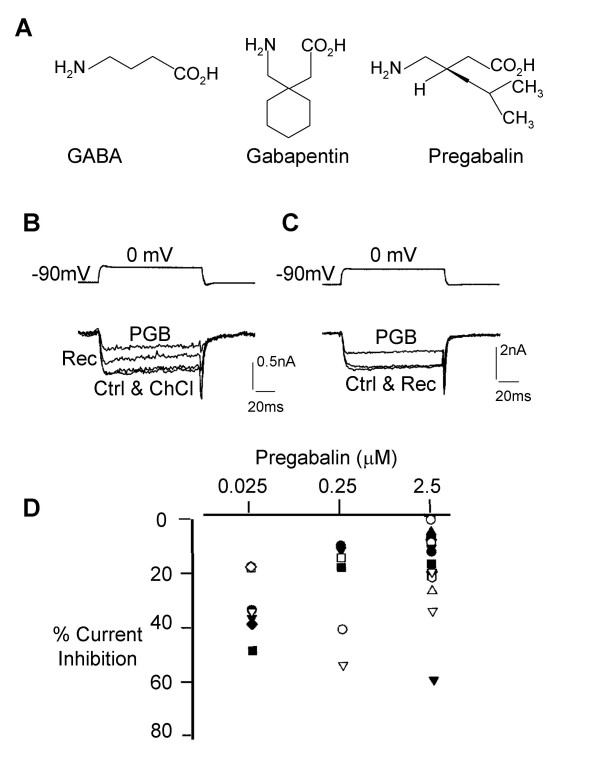
Pregabalin inhibits Ca^2+ ^currents. A) Structure of GABA (γ-aminobutyric acid), gabapentin (1-(aminoethyl)cyclohexane acetic acid) and pregabalin (S(+)-3-isobutyl GABA). B & C) Traces of high voltage-activated Ca^2+ ^currents evoked from a holding potential of -90 mV by a depolarising step command to 0 mV showing inhibition by pregabalin (2.5 μM). B) Shows that pressure ejection of choline chloride extracellular solution does not induce inhibition of the Ca^2+ ^current but in the same neurone pregabalin does produce a response. Traces show a control Ca^2+ ^current (Ctrl), the current unaffected by application of choline chloride recording solution (ChCl), inhibition of current by 3 minutes application of pregabalin (PGB) and the current at 5 minutes recovery (Rec). C) Traces show a control Ca^2+ ^current (Ctrl), the inhibition of current after 3 minutes application of pregabalin (PGB) and full recovery of the current 5 minutes after removal of the drug pipette (Rec). D) Graph showing the distribution of inhibitory responses produced by 0.025 – 2.5 μM pregabalin. Each symbol represents a result from a different experiment.

Gabapentin (Neurotonin^®^) and pregabalin (S(+)-3-isobutyl GABA) were both originally designed as GABA mimetics (Figure 1A), with the intention that they would be able to cross the blood-brain barrier and interact with GABAergic systems and enhance GABA mediated inhibition. Although gabapentin appears to have diverse therapeutic utility in the treatment of pain disorders [1], psychiatric illnesses [2] and epilepsy [3], there is controversy regarding its molecular mechanisms of action. Whether the actions of gabapentin and pregabalin are mediated through GABAergic mechanisms or GABA receptors remains particularly contentious [4-6]. However, high affinity binding sites for gabapentin and pregabalin on distinct α_2_δ subunits of voltage-activated calcium channels have been identified and characterised [7]. For this reason voltage-activated Ca^2+ ^channels remain primary candidate sites of action for these novel anticonvulsant and antihyperalgesic drugs. Functional data from studies on gabapentin and pregabalin also support this contention. Specifically, both gabapentin and pregabalin inhibited hyperalgesia [8,9], attenuated evoked Ca^2+ ^influx into brain slices and reduced evoked transmitter release [10]. Additionally, gabapentin and pregabalin inhibited multiple firing of action potentials evoked by 300 ms depolarising current commands in cultured sensory DRG neurones [5].

Our previous studies have focused on the inhibitory effects of gabapentin (0.25–25 μM) on whole cell voltage-activated Ca^2+ ^currents and K^+ ^stimulated Ca^2+ ^entry measured with fura-2. The cellular model systems used were primary cultures of dorsal root ganglion (DRG) neurones from 1–4 day old rats and differentiated F-11 cells (embryonic rat DRG × neuroblastoma hybrid cell line) [11,12].

In this present study the effects of pregabalin and gabapentin on the electrophysiological properties of cultured neonatal rat DRG neurones were measured with particular reference to Ca^2+ ^entry through high voltage-activated channels and enhancement of K^+ ^conductances. We had two specific aims for this project. The first was to determine whether gabapentin and pregabalin have the same mechanisms of action, by examining whether saturating concentrations of gabapentin and pregabalin act in an additive manner to attenuate Ca^2+ ^influx. The second aim was to further evaluate GABA receptors as target sites for these drugs in sensory neurones. This latter element to the study was conducted because it has been proposed that specific GABA_B _receptors with a gb1a-gb2 heterodimer composition are the sites of agonist activity of gabapentin. These specific receptors can be coupled to an inwardly rectifying K^+ ^channel and / or voltage-activated Ca^2+ ^channels to dampen neuronal electrical excitability [13-15]. However, in certain situations gabapentin and the GABA_B _receptor agonist, baclofen, have different actions. *Weaver *mutant mice (wv/wv) are insensitive to both gabapentin and baclofen, however in control littermates, *Weaver *control mice (+/+, wv/+), only baclofen evoked a K^+ ^current [16].

## Results and discussion

### Actions of pregabalin on voltage-activated calcium currents

The firing of multiple action potentials in response to a sustained depolarising current command is a property of a sub-population (under 20 %) of cultured DRG neurones. Application for 3 minutes, of either pregabalin (PGB; 2.5 μM) or gabapentin (GBP; 2.5 μM) reduced the frequency of action potential spikes evoked by 300 ms depolarising current step commands applied every 30 s [5]. Gabapentin attenuated repetitive action potential firing as measured by a reduction in the mean number of evoked action potentials during 300 ms depolarisations from 7 to 2 (n = 3, *p < 0.01*). Pregabalin (2.5 μM) reversibly reduced the number of action potentials during 300 ms depolarisations from 8 to 3 (n = 5, *p < 0.01*). However, pregabalin and gabapentin did not significantly alter the properties (amplitude, duration & threshold) of single action potentials evoked by 5 ms depolarising current commands.

Consistent with our previous work [11,12], whole cell voltage-activated Ca^2+ ^currents (I_Ca_) recorded from DRG neurones were reversibly attenuated (22 ± 11%; n = 7) by 2.5 μM gabapentin. Similarly, 3 minutes application of pregabalin (25 nM – 2.5 μM; Figure 1B &1C; table 1) inhibited the mean Ca^2+ ^current amplitude, measured at the peak of the inward current, and at the end of a 100 ms voltage step command to 0 mV. The inhibitory actions of pregabalin were not accompanied by any shift in the voltage-dependence of activation for I_Ca _or by any change in holding or leak currents. At least partial recovery of I_Ca _was observed 5 minutes after removal of the pregabalin-containing perfusion pipette (Figure 1B &1C). A very low concentration of pregabalin (0.25 nM) failed to produce significant inhibition of I_Ca_. However, no clear dose-dependent relationship was established for the inhibitory actions of pregabalin, and a major part of the voltage-activated Ca^2+ ^current was insensitive to the drug. It was also apparent that there was considerable variability in the sensitivity of the DRG neurones to any given dose of pregabalin. Some neurones did not respond to 2.5 μM pregabalin while in a few neurones this same concentration produced 60 % inhibition of I_Ca _(Figure 1D).

**Table 1:** Inhibitory actions of pregabalin on voltage-activated calcium currents.

Peak control current amplitude (nA)	Pregabalin Concentration (μM)	Peak current in the presence of pregabalin (nA)	Percentage inhibition and n value
-1.52 ± 0.13	0.025	-0.98 ± 0.12 **	31 ± 4 % (n = 8)
-1.2 ± 0.14	0.25	-0.93 ± 0.18 *	22 ± 9 % (n = 6)
-1.19 ± 0.11	2.5	-0.94 ± 0.09***	21 ± 3 % (n = 26)

***P < 0.001; **P < 0.01 and *P < 0.05. Each value is given ± S.E.M. Data shows inhibition but a lack of dose-dependent actions on I_Ca _of a wide range of pregabalin concentrations. Although varied responses were observed it was clear that a major component of I_Ca _was resistant to inhibition by pregabalin.

**Figure 2:**
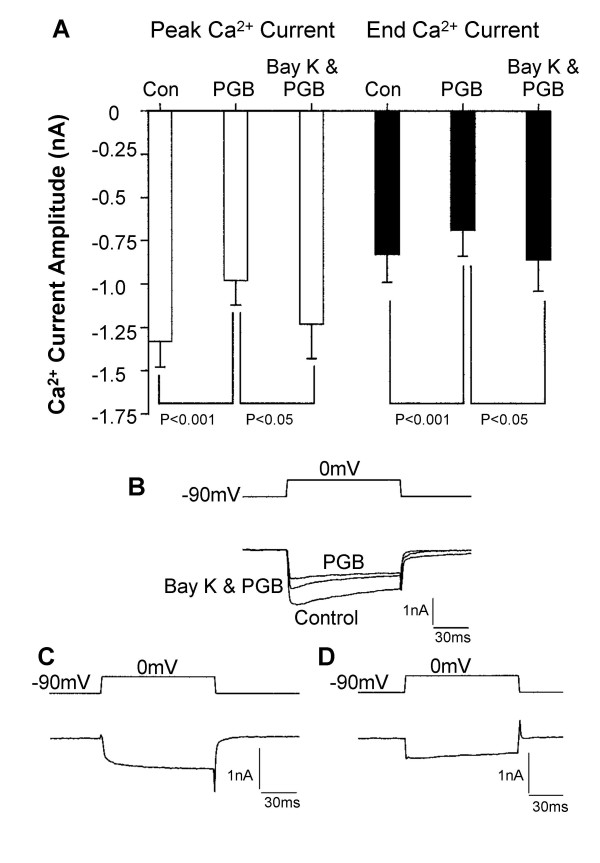
Bay K8644 enhanced pregabalin-insensitive current suggesting that L-type Ca^2+ ^channels are still available for modulation by the 1,4-dihydropyridine agonist. A) Bar chart shows data for mean calcium current amplitudes measured at the peak of the inward current (open bars) and at the end of a 100 ms voltage step command to 0 mV (solid bars, n = 6). Data under control condition (Con) in the presence of 2.5 μM pregabalin (PGB) and in the presence of both pregabalin (2.5 μM) and Bay K8644 (1 μM) are shown. B) The inset traces show voltage and Ca^2+ ^current records under control conditions, inhibition in the presence of 2.5 μM pregabalin (PGB) and enhancement of the Ca^2+ ^current during continued application of pregabalin with Bay K8644 present (Bay K & PGB). C) Shows the current inhibited by pregabalin, (obtained by subtracting the net current recorded in the presence of pregabalin from the net control current). D) Shows the additional current produced by Bay K8644, (obtained by subtracting the net current recorded in the presence of pregabalin from the net current recorded in the presence of both pregabalin and Bay K8644).

Distinct voltage-activated Ca^2+ ^channels, defined by their alpha 1 subunit, have been suggested to be selective target sites for gabapentin and related drugs like pregabalin. With this in mind, the 1,4-dihydropyridine L-type Ca^2+ ^channel agonist Bay K8644 was used to determine whether pregabalin was inhibiting L-type channels. Previously, we found that in the continual presence of gabapentin, Bay K8644 enhanced I_Ca _[12]. In this present study similar results were obtained. After inhibiting part of the current with pregabalin (2.5 μM), Bay K8644 (1 μM) was applied with pregabalin and the enhanced I_Ca _was measured at its peak and at the end of a 100 ms voltage step command to 0 mV (Figure 2). The percentage increases in current seen with Bay K8644 in the presence of either gabapentin or pregabalin were slightly less than those seen under control conditions in cultured DRG neurones [17]. This is consistent with some L-type Ca^2+ ^current modulation by pregabalin and gabapentin in DRG neurones. Taken together these data suggest that neither gabapentin nor pregabalin are selectively inhibiting L-type I_Ca _in DRG neurones. This contrasts with the effects of gabapentin on cortical pyramidal neurones where inhibition of L-type Ca^2+ ^channels appears to be the predominant mechanism of action [18].

### Actions of pregabalin on calcium influx through voltage-activated channels, measured using fura-2 imaging

**Figure 3:**
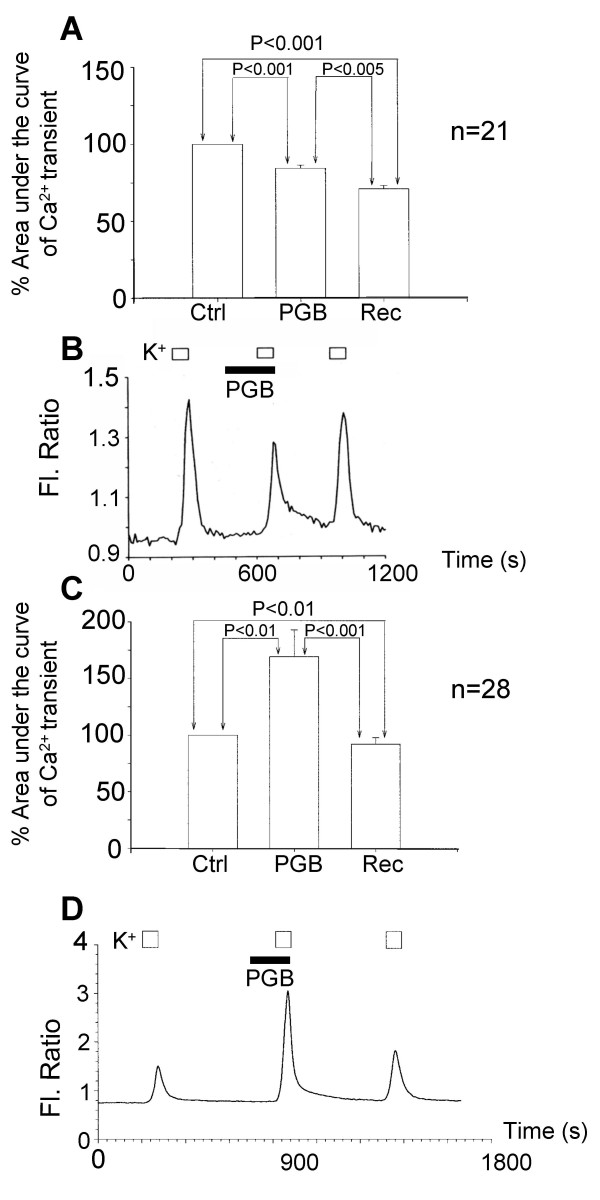
Pregabalin produced mixed actions on Ca^2+ ^influx evoked by 30 mM K^+^. A) Bar chart showing inhibition of Ca^2+ ^influx by pregabalin (25 μM). Data for the total Ca^2+ ^fluxes was normalised with respect to the first control response to K^+ ^(Ctrl). The second and third responses were obtained in the presence of pregabalin (PGB) and after washing away the pregabalin (Rec). B) Record of K^+^-evoked Ca^2+ ^transients showing partially reversible inhibition produced by 25 μM pregabalin (PGB). The period of stimulation is shown with open bars and PGB application with the filled bar. C) Bar chart showing enhancement of Ca^2+ ^influx by pregabalin (25 μM). Data for the total Ca^2+ ^fluxes were normalised with respect to the first control response to K^+ ^(Ctrl). The second and third responses were obtained in the presence of pregabalin (PGB) and after washing away the pregabalin (Rec). D) Record of K^+^-evoked Ca^2+ ^transients showing reversible enhancement produced by 25 μM pregabalin (PGB).

Given the variable and rather modest but reversible inhibitory actions of pregabalin on I_Ca _we tested the actions of pregabalin on K^+^-evoked Ca^2+ ^influx using fura-2 imaging. An extracellular solution containing 30 mM K^+ ^was used to depolarise the DRG neurones and activate three consistent Ca^2+ ^transients [11,12]. Pregabalin (2.5 μM) was applied during the second K^+^-evoked depolarisation and it produced a mixture of reversible effects on the Ca^2+ ^transients. In 4 of 24 neurones the Ca^2+ ^flux was decreased by 54 ± 13 % (*p*<0.05) but in 20 neurones from the same cultures pregabalin evoked a mean increase in Ca^2+ ^flux to 185 ± 20 % (*p*<0.05) of the control. Raising the pregabalin concentration to 25 μM increased the proportion of inhibitory responses (n = 21 out of 49 neurones) but enhancement of Ca^2+ ^transients was still observed in the remaining 28 neurones (Figure 3). However, 250 μM pregabalin caused an increase in K^+^-evoked Ca^2+ ^influx in all neurones studied (n = 31). Although in some cells good recovery from pregabalin actions was observed (figure 3B) the inhibitory effect was often not associated with recovery. This may reflect long lasting effects of pregabalin. Run-down in some cells can not be completely ruled out but under control conditions the mean level of run-down of three K^+^-evoked Ca^2+ ^transients was only 8.4 ± 1.5 % (n = 39). Similarly, the mean control level of increase in K^+^-evoked Ca^2+ ^transients was only 7.2 ± 1.3 % (n = 35).

**Figure 4:**
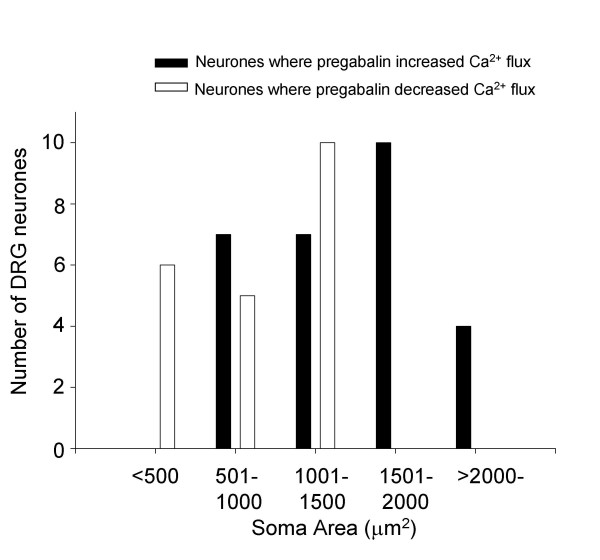
Different responses to pregabalin were observed in different populations of cultured DRG neurones. Bar chart showing the distribution of neurones with different cell soma areas and the response to pregabalin. The distributions for intermediate and larger neurones where pregabalin (25 μM) increased K^+^-evoked Ca^2+ ^influx are shown in black bars. The distributions for small and some intermediate neurones where pregabalin (25 μM) attenuated K^+^-evoked Ca^2+ ^influx are shown in open bars.

To investigate the different responses to pregabalin, the sizes of cell somas were measured and compared. Although there is overlap, figure 4 shows that enhancement of K^+^-evoked Ca^2+ ^transients by pregabalin was mainly seen in neurones with larger cell somas and that in smaller neurones pregabalin produced inhibitory effects.

**Figure 5:**
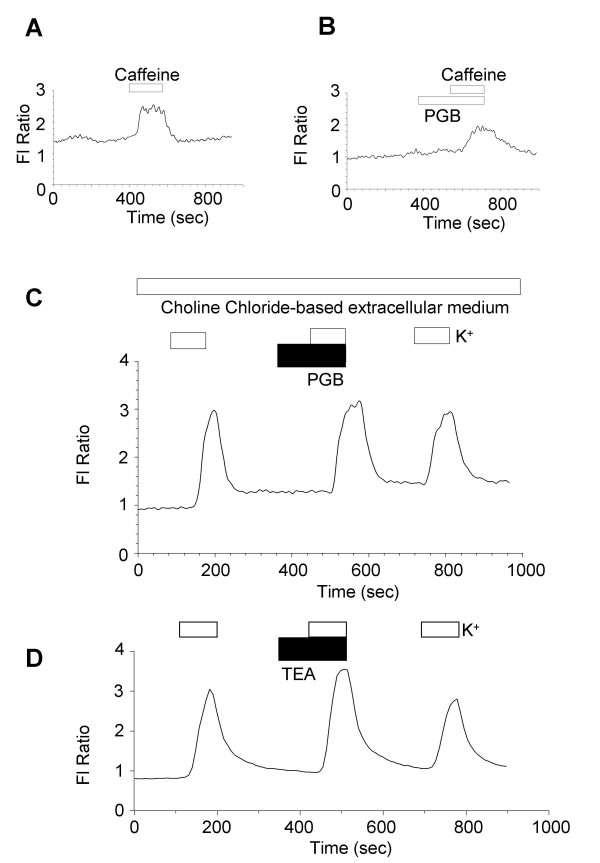
Pregabalin does not appear to modulate caffeine-evoked Ca^2+ ^release or Ca^2+ ^homeostatic mechanisms. A & B) Show example records of Ca^2+ ^transient evoked by caffeine (1 mM) applied to DRG neurones bathed in nominally Ca^2+^-free medium and measured using fura-2. Under these conditions the Ca^2+ ^transients are only due to mobilisation of Ca^2+ ^from intracellular stores. A) Illustrates a control caffeine response and B) shows a similar response to caffeine recorded in the presence of 25 μM pregabalin (PGB). C) Example trace showing an increase in K^+^-evoked Ca^2+ ^flux by 25 μM pregabalin (PGB) in a DRG neurone bathed with choline chloride-based extracellular medium. The periods of stimulation with 30 mM KCl are shown with open bars and pregabalin application is shown with a filled bar. D) Example trace showing an increase in K^+^-evoked Ca^2+ ^flux induced by 5 mM TEA in a DRG neurone bathed with standard NaCl-based extracellular medium. The periods of stimulation with 30 mM KCl are shown with open bars and TEA application is shown with a filled bar.

Ca^2+^-dependent conductances of DRG neurones are sensitive to ryanodine and Ca^2+^-induced Ca^2+ ^release has been reported in these neurones. Modulation of either Ca^2+^-induced Ca^2+ ^release and / or Ca^2+ ^homeostatic mechanisms might provide a mechanism by which pregabalin enhanced Ca^2+ ^transients in neurones with intermediate and large sized cell somas. This was investigated in two ways. Firstly, the actions of pregabalin on caffeine-evoked Ca^2+ ^transients were evaluated in nominally Ca^2+^-free extracellular conditions (NaCl-based solution with no added CaCl_2_). Single caffeine (1 mM) responses were obtained from DRG neurones in either the absence or presence of 25 μM pregabalin. No differences in either amplitudes or durations of Ca^2+ ^transients were seen when 8 control caffeine responses were compared with 4 caffeine responses obtained in the presence of pregabalin (Figure 5A,5B). The second approach was to explore a possible role of Na^+ ^/ Ca^2+ ^exchange by bathing cells in choline chloride-based medium containing only 1 mM Na^+^. Under these conditions the contribution of the Na^+ ^/ Ca^2+ ^exchanger to handling of intracellular Ca^2+ ^loads will be minimal. In choline chloride-based medium pregabalin produced both enhancement and inhibition of total Ca^2+ ^flux in 7 and 3 neurones respectively. Enhancement in Ca^2+ ^flux by pregabalin under these experimental conditions, suggest that inhibition of Na^+ ^/ Ca^2+ ^exchange is not the main mechanism by which pregabalin enhances K^+^-evoked Ca^2+ ^flux (Figure 5C). However, detailed analysis was made difficult by poor recovery of even the first Ca^2+ ^transient evoked in low extracellular Na^+ ^which does suggest that Na^+ ^/ Ca^2+ ^exchange is an important homeostatic mechanism in DRG neurones. In conclusion these results indicate that modulation of Ca^2+^-induced Ca^2+ ^release and Ca^2+ ^homeostatic mechanisms do not account for pregabalin-induced enhancement of K^+^-evoked Ca^2+ ^flux in a population of DRG neurones.

Inhibitory modulation of potassium conductances could result in increased K^+^-evoked Ca^2+ ^transients. To test this alternative mechanism, a relatively low concentration, 5 mM, of tetraethylammonium (TEA) was applied with NaCl-based extracellular medium and DRG neurones were stimulated with 30 mM KCl. Attenuation of potassium conductances by TEA markedly increased the K^+^-evoked Ca^2+ ^flux in all eight DRG neurones studied, mimicking in part the action of pregabalin (Figure 5D).

### Do pregabalin and gabapentin have additive effects on cultured dorsal root ganglion neurones?

**Figure 6:**
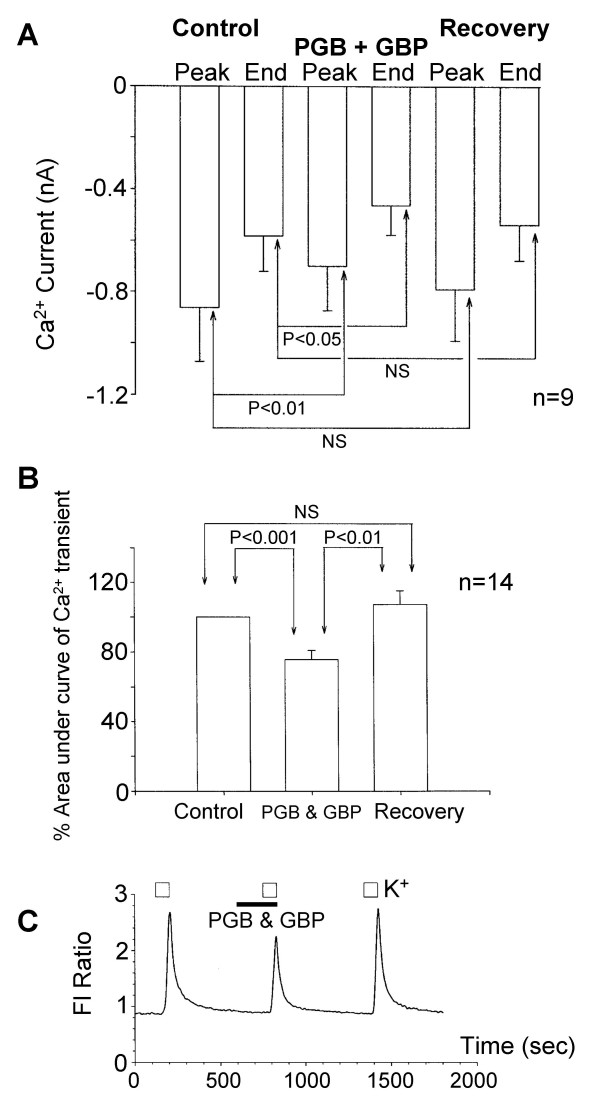
Pregabalin and gabapentin do not have additive actions on cultured DRG neurones. A) Bar chart showing the mean Ca^2+ ^current amplitude recorded at the peak of the inward current (Peak) and end of a 100 ms voltage step command (End). Data is shown for measurements made under control conditions (Control), after 3 to 5 minutes application of both pregabalin (2.5 μM) and gabapentin (2.5 μM) (PGB + GBP) and after 5 minutes recovery (Recovery). B) Bar chart showing normalised data from Ca^2+ ^imaging experiments in which simultaneous application of both pregabalin (25 μM) and gabapentin (25 μM) (PGB & GBP) reversibly attenuated the total Ca^2+ ^flux. C) Record of K^+^-evoked Ca^2+ ^transients, showing the reversible inhibition produced by 25 μM pregabalin and 25 μM gabapentin (PGB + GBP, filled bar).

Pregabalin and gabapentin have related chemical structures and appear to have similar but usually modest inhibitory effects on Ca^2+ ^currents. Both pregabalin and gabapentin at a concentration of 2.5 μM produced a maximum level of current inhibition. Simultaneous application of pregabalin (2.5 μM) and gabapentin (2.5 μM) produced modest but significant inhibition of I_Ca _at the peak of the current and at the end of the stimulus (Figure 6A). However, the percentage inhibition of I_Ca _produced was not significantly different for 2.5 μM gabapentin alone (22 ± 11%; n = 7), 2.5 μM pregabalin alone (21 ± 3%; n = 26) and 2.5 μM gabapentin and 2.5 μM pregabalin applied together (19 ± 2%; n = 9).

In spite of the added complexity of mixed responses with pregabalin, experiments were carried out using fura-2 to measure K^+ ^evoked Ca^2+ ^flux and the effects of both ligands. Our previous experiments with gabapentin (25 μM) had only identified inhibitory effects using this experimental approach [11,12]. Simultaneous application of pregabalin and gabapentin (both at 25 μM) reversibly reduced the K^+ ^evoked Ca^2+ ^influx (Figure 6B &6C). Pregabalin (25 μM) and gabapentin (25 μM) together reduced the total Ca^2+ ^flux to 75 ± 5 % (n = 14) of the control response to 30 mM K^+^. Consistent with the electrophysiological experiments, this level of inhibition was similar to the inhibitory effects of pregabalin and gabapentin applied separately.

The electrophysiological and fura-2 Ca^2+ ^imaging data show that no additive inhibitory effects were found during simultaneous application of saturating concentrations of gabapentin and pregabalin. The data therefore support the contention that both drugs have a closely related or a common site of inhibitory action. The data also indicate that in the modulation of Ca^2+ ^channels these drugs do not act in an additive manner even though a substantial proportion of current is resistant to both drugs. It should be emphasized however that pregabalin can reversibly enhance K^+^-evoked Ca^2+ ^transients, an effect not seen with gabapentin. Therefore there may be other additional actions of pregabalin that can be identified in Ca^2+ ^imaging experiments when all membrane conductances are intact.

### Does GABA receptor modulation alter responses to pregabalin in cultured dorsal root ganglion neurones?

**Figure 7:**
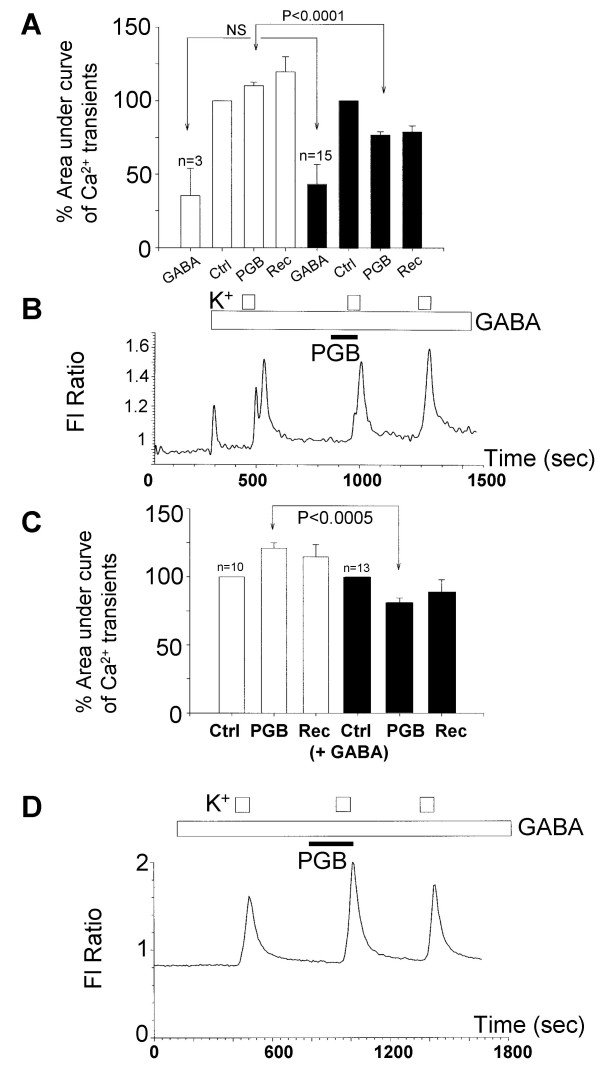
Activation and subsequent desensitisation of GABA receptors fails to prevent the modulation of voltage-activated Ca^2+ ^channels in cultured DRG neurones by pregabalin. A) Bar chart showing data obtained from neurones that responded to GABA (100 μM). All data are normalised with respect to the first Ca^2+ ^transient evoked by 30 mM KCl. Open bars show the relative responses to 100 μM GABA in cells (n = 3) where 25 μM pregabalin enhanced the Ca^2+ ^flux. Filled bars show responses in cells (n = 15) where 25 μM pregabalin inhibited the Ca^2+ ^flux. Data are shown for GABA responses (GABA), K^+^-evoked Ca^2+ ^transients under control conditions with GABA present (Ctrl), K^+ ^evoked Ca^2+ ^transients in the presence of GABA and 25 μM pregabalin (PGB) and on recovery in the continued presence of GABA (Rec). B) An example trace, showing a response to 100 μM GABA and Ca^2+ ^transients evoked by K^+ ^in the presence and absence 25 μM pregabalin. The long open bar shows the period of GABA application, the short open bars show K^+ ^stimulation and the filled bar the period of pregabalin application. C) Bar chart showing data obtained from neurones that did not responded to GABA (100 μM) but were continually bathed with GABA for the duration of the experiment. All data are normalised with respect to the first Ca^2+ ^transient evoked by 30 mM KCl. Data are shown for K^+^-evoked Ca^2+ ^transients under control conditions with GABA present (Ctrl), increased (n = 10; open bars) and decreased (n = 13; filled bars) K^+^-evoked Ca^2+ ^transients in the presence of GABA and 25 μM pregabalin (PGB) and on recovery (Rec). D) An example trace, showing no response to 100 μM GABA but responses to K^+ ^in the presence and absence of 25 μM pregabalin. The long open bar shows the period of GABA application, the short open bars show K^+ ^stimulation and the filled bar the period of pregabalin application.

In this section of the study two strategies were used to evaluate the possible roles of GABA receptors in pregabalin responses in cultured DRG neurones. Firstly, pregabalin actions were studied in the presence of a saturating concentration of GABA; this initially activated all types of GABA receptor and then caused desensitization of these receptors. Secondly, the effect of a potent and selective GABA_B _receptor antagonist, CGP52432 [19] on pregabalin responses was evaluated. GABA (100 μM) evoked inward currents in a sub-population of cultured DRG neurones and induced a transient rise in intracellular Ca^2+ ^in 18 of 41 DRG neurones studied (Figure 7A &7B). The inward currents were due to the activation of GABA_A _receptor Cl^- ^channels; with the equilibrium potential for Cl^- ^under our recording conditions being close to 0 mV the chloride conductance results in an inward current. Although *in vivo *the equilibrium potential for Cl^- ^in DRG neurones is variable, it is predicted to be between -40 mV and -20 mV because of Cl^- ^loading into the intracellular environment (for review see [20]). Therefore GABA_A _receptor Cl^- ^channel activation can produce a depolarisation of the resting membrane potential. Our data indicated that the GABA-evoked depolarisation was sufficient to result in voltage-activated Ca^2+ ^channel activity and produce a transient rise in intracellular Ca^2+^. In both neurones that responded to GABA and those that did not, subsequent application of pregabalin, produced either enhancement or inhibition of K^+^-evoked Ca^2+ ^influx (Figure 7A,7B,7C &7D; Table 2). These responses to pregabalin in the presence of GABA were no different in character to the pregabalin responses obtained in the absence of GABA. In neurones that showed a Ca^2+ ^transient in response to a GABA-evoked depolarisation, there was an apparent increase (to 83%) in the proportion of neurones that were inhibited by pregabalin. It is not clear why this is but it may reflect the sensitivity of Ca^2+ ^transients evoked in DRG neurones, which can only be evoked consistently in most DRG neurones by three depolarising stimuli. Thus the GABA-evoked responses may influence Ca^2+ ^homeostatic mechanisms and subsequent stimulated Ca^2+ ^entry. However, the critical observation from these experiments is that GABA receptor desensitisation does not prevent either Ca^2+ ^transient enhancement or inhibitory actions of pregabalin in neurones that were sensitive or insensitive to GABA.

**Table 2:** Actions of pregabalin (25 μM) on K^+^-evoked Ca^2+^influx in the continual presence or absence of 100 μM GABA.

GABA sensitivity	Mean percentage of control Ca^2+ ^influx	Number of neurones	Proportion of Neurones
Responders	110 ± 2 %	3 of 18	17 %
Responders	77 ± 2 %	15 of 18	83 %
Non-responders	121 ± 4 %	10 of 23	43 %
Non-responders	81 ± 3 %	13 of 23	57 %
Control	169 ± 24 %	28 of 49	57 %
Control	85 ± 2 %	21 of 49	43 %

Responders were those neurones in which GABA evoked a transient rise in intracellular Ca^2+^. Non-responder showed no change in fura-2 fluorescence ratio in response to GABA. Controls where neurones that pregabalin was applied to without GABA being present. The amplitudes of the GABA responses did not vary with the inhibitory or enhancing responses to pregabalin.

**Figure 8:**
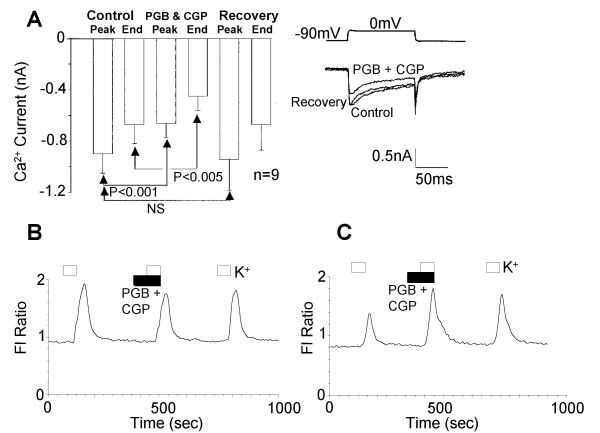
The GABA_B _receptor antagonist CGP52432 had no effect on the actions of pregabalin. A) Bar chart showing the inhibitory effects of 2.5 μM pregabalin in the presence of 10 μM CGP52432 (PGB & CGP) on Ca^2+ ^current amplitude measured at the peak inward current (Peak) and at the end of a 100 ms voltage step command to 0 mV (End). The inset traces show the inhibition of the Ca^2+ ^current produced by 3 minutes application of pregabalin and CGP52432 (PGB + CGP) and partial recovery 5 minutes after removal of the drug perfusion pipette. B & C) Show example records of 25 μM pregabalin in the presence of 10 μM CGP52432 (PGB + CGP) modulating Ca^2+ ^flux evoked by 30 mM KCl. The open bars show the period of stimulation with K^+ ^and the filled bar the application of pregabalin in the presence of CGP52432 (PGB +CGP).

The possibility of pregabalin-evoked inhibition of I_Ca _taking place through activation of G-protein coupled GABA_B _receptors, was then assessed. CGP52432 (10 μM) when applied alone had no effect on I_Ca_. In the presence of the GABA_B _receptor antagonist CGP52432 (10 μM), pregabalin (2.5 μM) evoked a 27 ± 3 % (n = 9) inhibition of the voltage-activated Ca^2+ ^current and complete recovery was observed 5 minutes after removal of the drug-containing pipette (Figure 8A). In Ca^2+ ^imaging experiments 10 μM CGP52432 had no effect on the responses to 25 μM pregabalin. In the presence of CGP52432, 4 out of 16 neurones showed enhanced K^+^-evoked Ca^2+ ^influx (to 161 ± 24 % (n = 4) of control) in response to pregabalin and 12 neurones showed inhibition of Ca^2+ ^influx (64 ± 8 % (n = 12) of control) in response to pregabalin (Figure 8B &8C).

### Is there a role for G-proteins or G-protein coupled receptors in the responses to pregabalin in cultured dorsal root ganglion neurones?

Previously, we found that the actions of gabapentin on I_Ca _were attenuated by pre-treating DRG neurones with pertussis toxin, an effect that did not appear to involve metabotropic GABA_B _receptors [12]. In this study several different approaches were taken to examine the influence of receptor and G-protein function in pregabalin actions. Firstly, the effects of a novel thiadiazole compound, SCH-202676, which inhibits ligand binding to a variety of G-protein coupled receptors (opioid, adrenergic, muscarinic and dopaminergic) were investigated. The selective and reversible action of SCH-202676 appears to involve allosteric modulation of both agonist and antagonist binding to G-protein coupled receptors [21]. Secondly, the potential influences of intracellular flash photolysis of caged GTP-γ-S and subsequent G-protein activation on pregabalin responses were also examined.

SCH-202676 (10 μM) was applied to the intracellular environment via the patch pipette solution. After 5 minutes equilibration the whole cell Ca^2+ ^current had a mean amplitude of -0.54 ± 0.08 nA (n= 7). During this period there was a clear reduction in the inward current, although a steady state current level was reached. However, subsequent application of pregabalin (2.5 μM) resulted in a further significant reduction in Ca^2+ ^current amplitude to -0.43 ± 0.07 nA (n = 7, *p*<0.05). This represents a mean inhibition of 22 ± 5 % by pregabalin, a value very similar to the percentage inhibition produced by pregabalin in the absence of SCH-202676 (21 ± 3%). Therefore SCH-202676 had no effect on the pregabalin responses, indicating that pregabalin was not acting through an SCH-202676-sensitive G-protein coupled receptor (data not shown). Preliminary Ca^2+ ^imaging experiments were also conducted to determine whether extracellular SCH-202676 (10 μM, continually applied throughout the experiment) altered the effects of pregabalin (2.5 μM) on K^+^-evoked Ca^2+ ^transients. Under these experimental conditions pregabalin was found to markedly enhance or inhibit K^+^-evoked Ca^2+ ^transients (data not shown). This data again suggests that pregabalin was not acting through mechanisms that were sensitive to SCH-202676.

**Figure 9:**
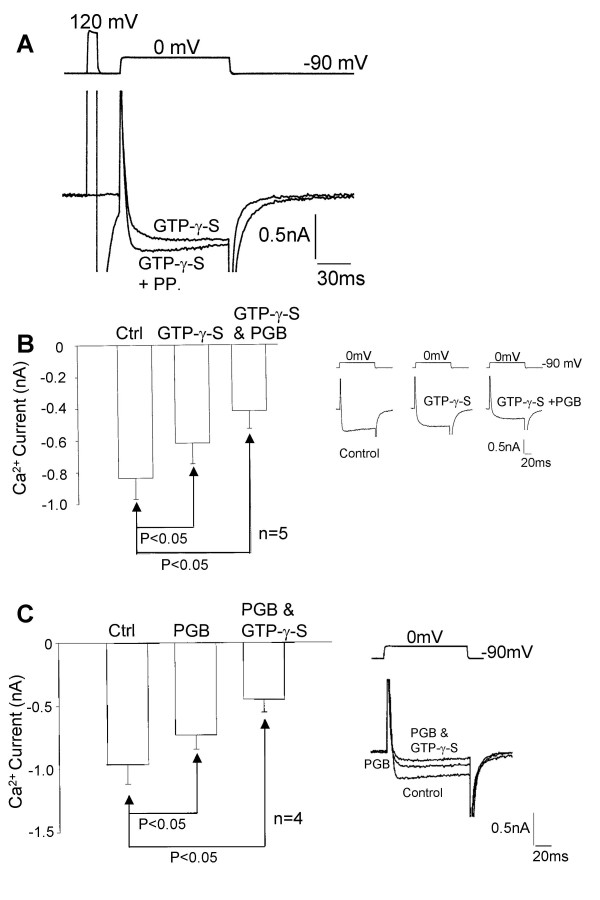
Intracellular photorelease of GTP-γ-S did not alter the sensitivity of DRG neurones to pregabalin. A) Traces showing a Ca^2+ ^current attenuated and slowed by intracellular flash photolysis of caged GTP-γ-S (GTP-γ-S) and a Ca^2+ ^current showing voltage-dependent partial recovery of GTP-γ-S-evoked inhibition (GTP-γ-S + PP {pre-pulse to +120 mV}). B) Bar chart showing control (Ctrl) data, the inhibitory effects of both intracellular photorelease of GTP-γ-S (GTP-γ-S) and subsequent application of 2.5 μM pregabalin (GTP-γ-S & PGB) on the mean peak Ca^2+ ^current amplitude. Inset records show individual Ca^2+ ^currents recorded under control conditions, after intracellular photorelease of GTP-γ-S (GTP-γ-S) and after subsequent extracellular application of pregabalin for 3 minutes (GTP-γ-S + PGB). C) Bar chart showing control (Ctrl) data, the inhibitory effects of both 3 minutes extracellular application of 2.5 μM pregabalin (PGB) and subsequent intracellular photorelease of GTP-γ-S in the continued presence of pregabalin (PGB & GTP-γ-S) on the mean peak Ca^2+ ^current amplitude. Inset records show individual Ca^2+ ^currents recorded under control conditions, after application of pregabalin for 3 minutes (PGB) and after intracellular photorelease of GTP-γ-S (PGB & GTP-γ-S). None of the currents in this figure have been leak subtracted. However, neither pregabalin nor GTP-γ-S altered the leak current.

Caged GTP-γ-S (100 μM) was applied to the intracellular environment via the patch pipette solution and after entering the whole cell recording configuration DRG neurones were left for 5 minutes to equilibrate. Once a stable control Ca^2+ ^current was obtained, three 200 V flashes of intense near UV light were applied to the neurone to achieve intracellular flash photolysis of the caged GTP-γ-S. We estimate that approximately 15 μM GTP-γ-S was photoreleased by the three flashes. As previously observed intracellular flash photolysis of caged GTP-γ-S reduced the amplitude and slowed the activation of I_Ca _[22]. The effects of photoreleased GTP-γ-S were attenuated by applying a large depolarising pre-pulse [23], which is consistent with voltage-dependent G-protein modulation of Ca^2+ ^channels (Figure 9A). No recovery from photoreleased GTP-γ-S was observed during the period of the experiment. GTP-γ-S and pregabalin (2.5 μM) had additive inhibitory effects on I_Ca_. These additive actions of GTP-γ-S and pregabalin were apparent regardless of the order of application. One set of experiments was performed in which GTP-γ-S was photoreleased in the intracellular environment and then after stabilisation of the response pregabalin was applied (Figure 9B). In another set of experiments pregabalin was applied first and after equilibration GTP-γ-S was photoreleased in the continual presence of pregabalin (Figure 9C). When pregabalin (2.5 μM) was applied first it produced a mean inhibition in I_Ca _by 25 ± 5 % (n = 4), when applied after photorelease of GTP-γ-S, pregabalin produced a 20 ± 10 % (n = 5) inhibition of I_Ca_.

### Actions of pregabalin on voltage-activated potassium currents

It was clear from the Ca^2+ ^imaging experiments that not all the cellular actions of pregabalin could be explained by the inhibition of voltage-activated Ca^2+ ^channels because both inhibition and enhancement in K^+ ^evoked Ca^2+ ^flux was observed. To investigate the actions of pregabalin further, its' effects on voltage-activated K^+ ^currents in DRG neurones were studied. Previously, Stefani and colleagues found that gabapentin modulated neuronal steady state non-inactivating K^+ ^currents [18].

**Figure 10:**
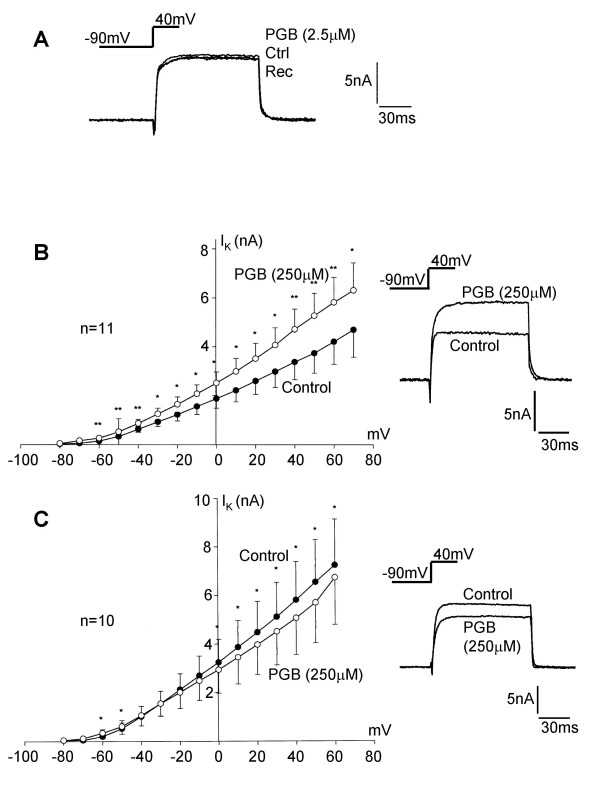
Pregabalin modulates voltage-activated potassium currents in cultured DRG neurones. A) Traces showing that 2.5 μM pregabalin failed to significantly alter the outward K^+ ^current evoked by a 100 ms voltage step command from -90 mV to 40 mV. B) Current / voltage relationship showing enhancement of outward current following 3 minutes application of 250 μM pregabalin (filled circles = control data and open circles currents recorded in the presence of pregabalin (PGB); * = P < 0.05 & ** = P < 0.01). Inset traces show the control outward K^+ ^current activated at +40 mV and the enhanced K^+ ^current recorded in the presence of pregabalin (250 μM). C) Current / voltage relationship showing inhibition of outward K^+ ^current following 3 minutes application of 250 μM pregabalin (filled circles = control data and open circles K^+ ^currents recorded in the presence of pregabalin (PGB); * = P < 0.05). Inset traces show the control outward K^+ ^current activated at +40 mV and the attenuated K^+ ^current recorded in the presence of pregabalin (250 μM).

Three minutes application of pregabalin (2.5 μM) had no significant effect on the voltage-activated K^+ ^current activated from a holding potential of -90 mV by a 100 ms voltage step command to 0 mV (Figure 10A). However, raising the pregabalin concentration to 250 μM resulted in significant modulation of K^+ ^currents in DRG neurones. Pregabalin (250 μM) applied for 3 to 5 minutes produced an enhanced K^+ ^current in 11 out of 21 neurones but a modest inhibition of the outward current in 10 out of 21 neurones (Figure 10B and 10C). DRG neurones are a heterogenous population of neurones and there is evidence that they express at least 6 diverse voltage-activated K^+ ^channels and that this expression is in part determined by the type of DRG neurone [24]. To distinguish between the two responses experiments were carried out on DRG neurones held at -30 mV to inactivate a proportion of the outward current. Under these conditions pregabalin inhibited the outward current in 9 out of 11 DRG neurones but still produced enhancement in 2 neurones. The level of inhibition increased at a holding potential of -30 mV to 30 ± 7% (n = 9) compared to 15 ± 4 % (n = 10) at -90 mV, (data not shown).

**Figure 11:**
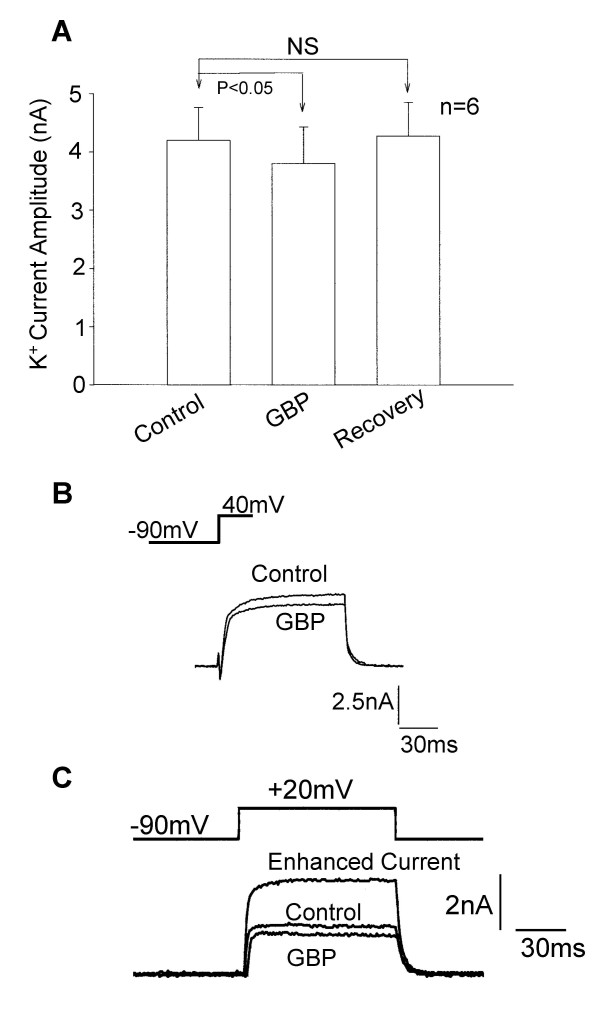
Acute application of gabapentin produced modest inhibition of voltage-activated K^+ ^currents in cultured DRG neurones but long-term measurement of K^+ ^currents shows a delayed enhancement in outward current. A) Bar chart showing data for the acute (3–5 minutes) reversible inhibition of the mean K^+ ^current by 250 μM gabapentin (GBP). B) Traces showing a control outward K^+ ^current activated at 0 mV and the inhibited current activated at the same voltage after 3 minutes application of 250 μM gabapentin (GBP). C) Traces showing the biphasic response to 250 μM gabapentin. Illustrated are the control current, the inhibited current recorded after 5 minutes application of gabapentin (GBP) and the enhanced outward current measured 5 minutes after removal of the perfusion pipette containing gabapentin (Enhanced Current).

Interestingly, in apparent contrast to pregabalin, 250 μM gabapentin was initially only found to inhibit K^+ ^currents in DRG neurones (Figure 11A,11B). However, when studies into long-term (10–15 minutes) actions of gabapentin were investigated slowly developing outward current enhancement was identified. So similar to actions of pregabalin, biphasic responses to gabapentin were found with initial inhibition of K^+ ^currents and then a delayed enhancement of the outward current (Figure 11C), [5].

**Figure 12:**
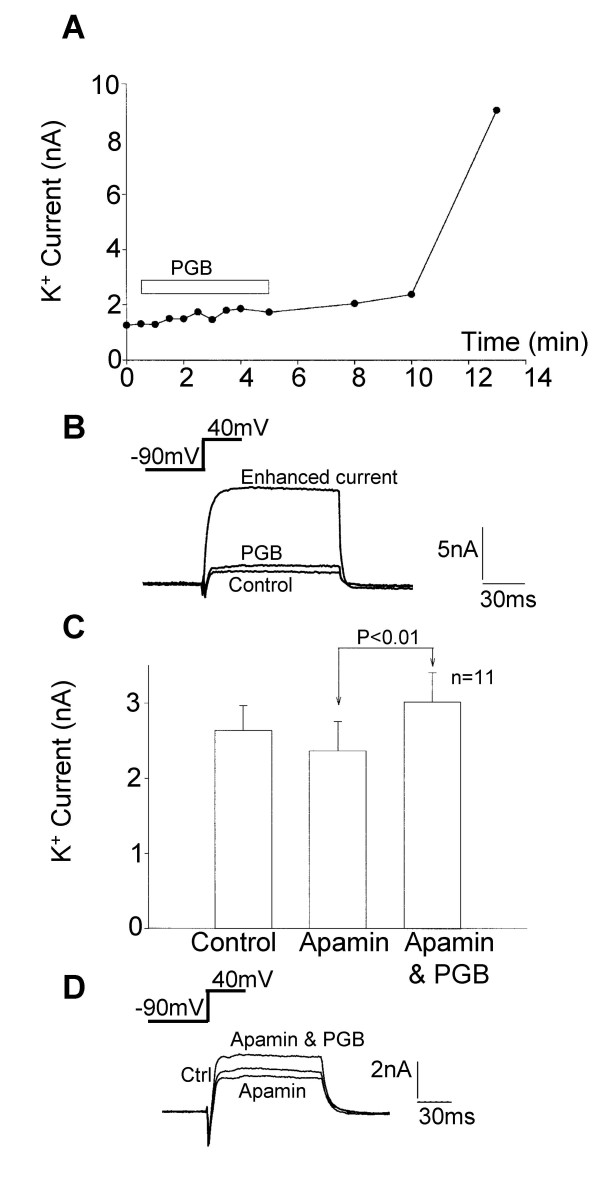
Pregabalin produced delayed enhancement of K^+ ^currents in cultured DRG neurones. A) Line graph showing a time course for the delayed action of pregabalin in a single neurone, the open bar shows the period of application of pregabalin (250 μM). Little effect of pregabalin was seen in this neurone until 5 minutes after removal of the pressure ejection pipette containing pregabalin. B) Inset traces show a control K^+ ^current activated at +40 mV, modest enhancement of the K^+ ^current after 5 minutes application of 250 μM pregabalin and the enhanced outward K^+ ^current recorded 9 minutes after application of pregabalin. Apamin mimicked the inhibitory action of pregabalin on K^+ ^current but did not prevent enhancement of the K^+ ^current by acute application of 250 μM pregabalin. C) Bar chart showing the modest inhibitory effect of apamin (1 μM) on K^+ ^current and the enhancement in K^+ ^current when pregabalin was applied in the continued presence of apamin (Apamin & PGB). D) Traces showing a control K^+ ^current, the inhibited K^+ ^current in the presence of 1 μM apamin and the enhanced K^+ ^current observed when apamin and pregabalin were applied together. Apamin prevented the inhibitory effect of pregabalin but not the enhancement of K^+ ^current.

Long-term modulation of K^+ ^current by pregabalin was then investigated. Dramatic increases in outward current were observed after a delay. This effect of pregabalin persisted even as pregabalin was removed from the extracellular environment, which may implicate a metabolic or intracellular signalling event in this response. The mean K^+ ^current amplitude at +40 mV increased from 3.37 ± 0.73 nA to 7.56 ± 1.10 nA (n = 11; *p*<0.01) 15 minutes after perfusion of 250 μM pregabalin. Figures 12A and 12B show an individual example record and trace of the delayed response to pregabalin. These responses did reverse but this took about 40 minutes with the response developing 3–10 minutes after the start of pregabalin application. No change in holding current or in the leak conductances were associated with the long-term effect of pregabalin and in the absence of pregabalin stable K^+ ^currents were recorded from DRG neurones for 16 minutes (n = 10).

Several pharmacological experimental approaches were taken to characterise the biphasic actions of pregabalin and to determine the possible mechanism of action associated with the long-term K^+ ^current modulation. Apamin, a toxin from the honeybee, was used to block small conductance Ca^2+^-activated K^+ ^currents. Apamin (1 μM) caused a reduction in outward current at +40 mV from 2.64 ± 0.33 nA to 2.37 ± 0.39 nA, subsequent application of 250 μM pregabalin enhanced the current to 3.02 ± 0.39 nA (n = 11; *p*<0.01). No inhibitory effects were seen with pregabalin after treatment with apamin, suggesting that it is the apamin-sensitive Ca^2+^-activated K^+ ^channels that are inhibited by pregabalin (Figure 12C &12D).

**Figure 13:**
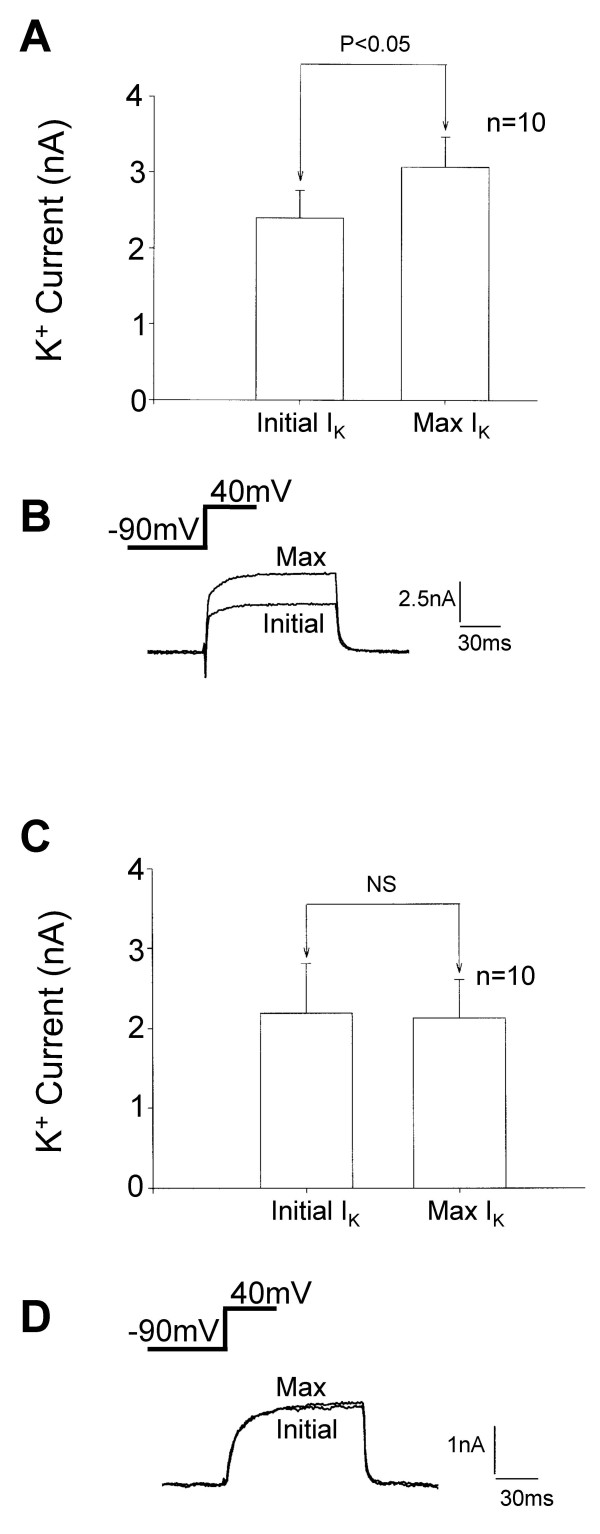
Intracellular application of pregabalin enhanced K^+ ^currents in cultured DRG neurones. For these experiments pregabalin was included in the KCl-based patch pipette solution at a concentration of 250 μM. A) Bar chart showing the mean amplitudes of the K^+ ^current recorded immediately after entering the whole cell recording configuration (Initial I_K_) and the maximum outward current recorded within 16 minutes of entering the whole cell recording configuration (Max I_K_). B) Traces of the first K^+ ^current recorded using a patch pipette solution containing 250 μM pregabalin (Initial) and from the same cell the maximum outward K^+ ^current recorded with intracellular pregabalin (Max). C) Bar chart showing control data recorded from neurones not exposed to pregabalin. Illustrated are the mean amplitudes of the K^+ ^current recorded immediately after entering the whole cell recording configuration (Initial I_K_) and the maximum K^+ ^outward current recorded within 16 minutes of entering the whole cell recording configuration (Max I_K_). D) Traces of the first K^+ ^current recorded using the standard KCl-based patch pipette solution (Initial) and from the same cell the maximum outward current recorded under control conditions, within 16 minutes of entering the whole cell recording configuration (Max).

Gabapentin can be transported into cells via the L α-amino acid transporter and may achieve intracellular concentrations 10–20 times the levels in the extracellular environment [25]. Furthermore, actions through intracellular signalling pathways and specifically protein kinase A, have been proposed for gabapentin [12]. To examine whether the long-term enhancement of K^+ ^currents by pregabalin might involve intracellular sites of action, we applied pregabalin to the intracellular environments of DRG neurones via the patch pipette solution. Intracellular pregabalin (250 μM) evoked an increase in the K^+ ^current that started to develop within the first minute of entering the whole cell recording configuration. After an initial increase that could reflect equilibration with the KCl-based patch pipette solution containing pregabalin, a slow sustained significant increase in the outward K^+ ^current continued to develop over a 15 minute period (n = 10; *p*< 0.01; Figure 13A,13B). No similar change in K^+ ^current was observed in control studies carried out in the absence of pregabalin (Figure 13C,13D).

**Figure 14:**
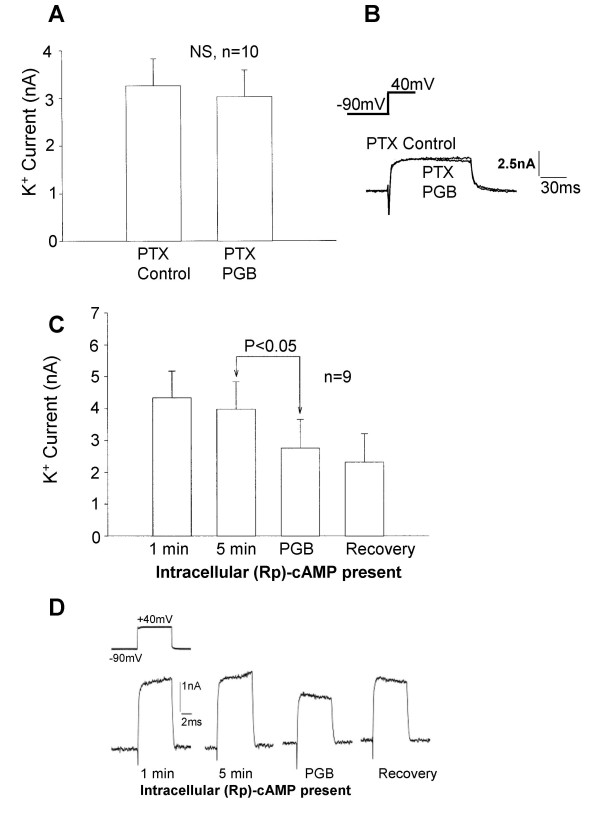
Pertussis toxin pre-treatment and intracellular (Rp)-cAMP prevented enhancement of K^+ ^current by pregabalin. A) Bar chart showing the mean amplitude of K^+ ^current recorded from DRG neurones pre-treated with pertussis toxin for 16–18 hours with 500 ng/ml (PTX Control) and after application of 250 μM pregabalin (PTX PGB), long term, up to 15 minutes monitoring of the current. B) Traces showing outward K^+ ^currents recorded from a DRG neurone pre-treated with pertussis toxin, prior to pregabalin application (PTX Control) and 10 minutes after application of 250 μM pregabalin. C) Bar chart showing mean data obtained from neurones containing (Rp)-cAMP (30 μM), which was applied to the intracellular environment via the patch pipette solution. Data shows the mean K^+ ^current amplitude recorded 1 minute and 5 minutes after entering the whole cell recording configuration (1 min; 5 min), after 5 minutes application of 250 μM pregabalin (PGB) and 10 minutes after removal of the pressure ejection pipette containing pregabalin. D) Traces from a single experiment showing the outward K^+ ^currents at 1 and 5 minutes after entering the whole cell recording configuration and allowing entry of 30 μM (Rp)-cAMP in the DRG neurones. Also shown are the K^+ ^current inhibited by 5 minutes application of 250 μM pregabalin (PGB) and the recovery of the K^+ ^current after the pressure ejection pipette containing pregabalin was removed. Intracellular (Rp)-cAMP prevented the delayed long-term enhancement of the K^+ ^current evoked by pregabalin.

Pertussis toxin pre-treatment results in the uncoupling of sensitive G-proteins from effector mechanisms, including voltage-activated Ca^2+ ^and K^+ ^channels. Additionally, pertussis toxin pre-treatment has previously been found to influence gabapentin actions on I_Ca_. Pertussis toxin pre-treatment (500 ng/ml; 16–18 hours) prevented pregabalin-induced long-term K^+ ^current enhancement in cultured DRG neurones (Figure 14A &14B).

The possible role of cAMP-dependent protein kinase A (PKA) in the pregabalin-induced enhancement of K^+ ^current was then assessed using the inhibitor (Rp)-cAMP [26]. Intracellular application of 30 μM (Rp)-cAMP applied via the KCl-based patch pipette solution had no effect over a 5 minute equilibration period on voltage-activated K^+ ^current (n = 9). This may indicate that PKA has little or no basal or tonic activity on K^+ ^currents in DRG neurones in culture. However, when pregabalin was applied to DRG neurones loaded with (Rp)-cAMP no long-term enhancement of the outward current was observed. Under these recording conditions pregabalin still produced some inhibition of the K^+ ^current (Figure 14C &14D). These data provide evidence that the long-term modulation of voltage-activated K^+ ^channels by pregabalin is dependent on PKA-mediated phosphorylation.

## Conclusions

In conclusion, these results indicate that pregabalin acts via the same basic mechanisms as gabapentin to inhibit voltage-activated Ca^2+ ^channels and that these inhibitory actions are independent of GABA receptor activation. Some features of the actions of pregabalin on intermediate size and large DRG neurones appear not to be seen with gabapentin. However, these distinct responses involve enhanced K^+^-evoked Ca^2+ ^transients by pregabalin rather than inhibition of Ca^2+ ^channels.

Alpha_2_δ subunits of voltage-activated Ca^2+ ^channels remain a possible site of action for both pregabalin and gabapentin. Dooley and colleagues showed that both gabapentin and pregabalin attenuated K^+^-evoked norepinephrine release from rat neocortical slices by inhibiting P/Q-type Ca^2+ ^channels [10]. In cortical pyramidal neurones gabapentin predominantly works through L-type Ca^2+ ^channels [27]. Our work with Bay K8644 indicates that in cultured DRG neurones gabapentin [5] and pregabalin act predominantly independently of L-type channels. A recent study has also indicated that acting via G-protein coupled GABA_B _receptors, gabapentin selectively inhibited N-type Ca^2+ ^channels in hippocampal pyramidal neurones [15]. Interestingly, this selectivity of gabapentin seen in the hippocampus is different from the Ca^2+ ^channel modulation seen with the GABA_B _receptor agonist, baclofen. Our previous investigation using "toxityping" showed that gabapentin inhibited a variety of Ca^2+ ^channels in DRG neurones [11]. We conclude from all this work that gabapentin and pregabalin may have allosteric interactions with promiscuous α_2_δ Ca^2+ ^channel subunits. These α_2_δ subunits are not specifically combined with distinct pore forming Ca^2+ ^channel subunits (α_1_) in all neurones and may therefore inhibit pharmacologically diverse Ca^2+ ^channels depending on the expression of Ca^2+ ^channel subunits in different neurones. However, transfection studies have shown that oocytes expressing Ca^2+ ^channels (Ca_v_2.2) containing β1b and α_2_δ-1 or α_2_δ-2 subunits are insensitive to acute application of 50 μM gabapentin [28]. This work raises the possibility that other mechanisms independent of α_2_δ – Ca^2+ ^channel subunits are involved in the modulation of neuronal excitability by gabapentin. Additionally, the modulation of voltage-activated Ca^2+ ^channels by gabapentin and pregabalin acting through indirect mechanisms has been suggested. Candidates for such indirect mechanisms include the control of Ca^2+ ^channel functional expression [29,30] and metabotropic mechanisms linked to pertussis toxin sensitivity and PKA activation [12].

In hippocampal pyramidal neurones, uncoupling G-proteins from metabotropic receptors with N-ethylmaleimide prevents modulation of K^+ ^and Ca^2+ ^channels by gabapentin [15]. However, in DRG neurones it is not clear how pertussis toxin influences the actions of gabapentin and pregabalin but it may involve disruption of multi-protein complexes of G-proteins and ion channel subunits after ADP-ribosylation of Gα. The role of any G-protein coupled receptors in either gabapentin or pregabalin responses in DRG neurones are not supported by our investigations with SCH-202676. Furthermore, receptor and direct G-protein involvement in pregabalin effects on Ca^2+ ^channels also appears unlikely in DRG neurones. This is because in this study we found that intracellular flash photolysis of GTP-γ-S had no influence on pregabalin-evoked current inhibition and pregabalin did not alter responses to GTP-γ-S. Our findings are in agreement with those made in a previous study on cells expressing GABA_B1a/B2 _or GABA_B1b/B2 _receptor subunits. In this study GABA and the GABA_B _receptor agonist baclofen evoked [^35^S]-GTP-γ-S binding responses but even at high concentrations both gabapentin and pregabalin did not [31].

The experiments designed to assess potential roles of GABA receptors in the responses to pregabalin showed that GABA receptor desensitization or the blockade of GABA_B _receptors did not attenuate pregabalin actions. These findings add to the published studies that indicate that gabapentin and pregabalin effects are independent of GABA receptor activation at least in some preparations [31,32]. Specifically, in cultured DRG neurones gabapentin was previously found not to activate a Cl^- ^conductance to alter membrane potential and input resistance and the GABA_B _receptor antagonist saclofen did not influence the inhibitory action of gabapentin on I_Ca _[12].

Pregabalin has a higher affinity for α_2_δ subunits than gabapentin and in a number of studies is more effective. When applied alone pregabalin produced enhancement in Ca^2+ ^flux in some neurones, an effect not seen with gabapentin. So, it was surprising to see in the imaging experiments that when gabapentin and pregabalin were applied together they produced only inhibitory effects. This may reflect the allosteric interactions between these drugs and α_2_δ subunits of Ca^2+ ^channels as well as indirect modulation of Ca^2+ ^dependent conductances and interactions with components of cell signalling.

There appear to be some inconsistencies in the pregabalin data when its actions on voltage-activated Ca^2+ ^currents are compared with effects on K^+^-evoked Ca^2+ ^transients. This may in part be due to effects of pregabalin on membrane conductances in addition to voltage-activated Ca^2+ ^currents. These effects may not be detected under voltage clamp recording conditions where Na^+ ^and K^+ ^currents are blocked to isolate Ca^2+ ^currents. In the imaging experiments all voltage-activated conductances are intact and may be modulated by pregabalin. Alternatively, pregabalin could potentially have a direct or indirect influence on Ca^2+^-induced Ca^2+ ^release from intracellular stores and alter Ca^2+ ^homeostatic mechanisms. These effects may be modulated differently in different sub-populations of DRG neurones and so produce the mixed responses recorded in small, intermediate and large neurones in this study. Thus different actions of pregabalin may be detected using fura-2 fluorescence imaging but these additional mechanisms may not influence the measurements of Ca^2+ ^currents. Although mixed responses to gabapentin were not previously identified, neither were clear effects on the amplitude of K^+^-evoked Ca^2+ ^transients apparent in DRG neurones [11], although they were seen in differentiated F-11 cells [12]. These apparent anomalies seen with pregabalin do not appear to be due to drug effects on Ca^2+^-induced Ca^2+ ^release or Ca^2+ ^homeostatic mechanisms such as Na^+^/Ca^2+ ^exchange. This conclusion was reached because pregabalin did not influence Ca^2+ ^transients evoked by caffeine and still enhanced K^+^-evoked Ca^2+ ^transients in choline chloride-based (low Na^+^) extracellular solution.

The mixed responses to pregabalin seen in the Ca^2+ ^imaging experiments prompted us to investigate the actions of pregabalin on voltage-activated K^+ ^currents, which could be modulated to cause an increase in Ca^2+ ^influx. This speculation was supported by the finding that application of the K^+ ^channel inhibitor, TEA, enhanced K^+^-evoked Ca^2+ ^transients. Stefani and colleagues have reported inhibition of outward K^+ ^currents by gabapentin [18]. Additionally, in rat hippocampal and human neocortical brain slices gabapentin has been shown to inhibit K^+^-evoked [^3^H]-noradrenaline release. The activation of K_ATP _channels is implicated in these responses because not only does glibenclamide, a K_ATP _channel antagonist, attenuate the gabapentin response but also pinacidil, a K_ATP _channel agonist, mimics the response and did not have additive effects with gabapentin [33].

Mixed or biphasic responses to gabapentin and pregabalin were seen, with both inhibition and enhancement of K^+ ^currents observed. The inhibitory actions of both gabapentin and pregabalin on K^+ ^currents recorded from DRG neurones may not reflect a direct action of these drugs on K^+ ^channels. The effects of apamin, which prevented the inhibition of outward current by pregabalin indicated that small conductance Ca^2+^-activated K^+ ^channels were involved in the response. This is consistent with the effects of gabapentin seen in isolated cortical neurones [18]. The pregabalin inhibitory action on voltage-activated Ca^2+ ^channels may underlie the inhibition of outward K^+ ^current. A reduction in Ca^2+ ^influx would lead to reduced activation of Ca^2+^-activated K^+ ^channels and therefore a smaller outward current. If this indirect effect of pregabalin resulted in a disproportionate reduction in Ca^2+^-activated K^+ ^conductances this may result in prolonged depolarisation. In turn a prolonged depolarisation may maintain activation of Ca^2+ ^channels that are insensitive to pregabalin and an increase in Ca^2+ ^influx as seen in a sub-population of larger DRG neurones in the Ca^2+ ^imaging experiments. It is not clear why gabapentin, which produced modest inhibition of K^+ ^currents, did not also produce enhancement of K^+^-evoked Ca^2+ ^transients. It may be explained by a balance between inhibition of Ca^2+ ^influx through voltage-activated channels and modulation of Ca^2+ ^flux through inhibition of K^+ ^conductance. Consistent with this hypothesis, gabapentin appears to be less effective than pregabalin at modulating K^+^-evoked Ca^2+ ^transients in DRG neurones. This is supported by our previous study that showed that gabapentin did not significantly alter the peak Ca^2+ ^transients [11].

The delayed enhancement of voltage-activated K^+ ^currents by gabapentin and pregabalin is a powerful mechanism for reducing cell excitability. The slow development of this response and its gradual decline suggested that intracellular signalling events were involved. Pregabalin produces enhancement of K^+ ^currents when applied either inside or outside DRG neurones. The delay in the development of these responses is less with intracellular application of pregabalin, suggesting that it may have an intracellular site of action. This effect may also depend on drug uptake into cells via L α-amino acid transporters and the presence and activity of these transporters may greatly influence cell sensitivity to gabapentin and pregabalin. Although it is not clear how gabapentin and pregabalin might activate PKA, it is a candidate target site. Our previous work showed that inhibition of Ca^2+ ^currents by gabapentin was sensitive to cAMP analogues that activated or inhibited PKA [12]. Similarly, in this present study (Rp)-cAMP blocked enhancement of K^+ ^currents by pregabalin. In the literature there are a number of reports of PKA altering neuronal excitability and modulating K^+ ^conductances. PKA is involved in pain signalling, playing a role in prostaglandin-induced activation and sensitization of DRG neurones [34]. PKA activity attenuates KV3.2 channel currents [35] and A-type current [36] and is involved in the inhibition of K^+ ^conductances by prostaglandin E-2 [37]. Our results are not explained by these findings but PKA has also been implicated in presynaptic inhibition of GABA release [38], large conductance calcium – and voltage-activated K^+ ^channel activity [39] and cannabinoid receptor mediated modulation of ion channels [40]. In the context of our project, of particular interest is the biphasic modulation in retinal cones of voltage dependent K^+ ^and Ca^2+ ^channels by a synthetic cannabinoid receptor agonist and the sensitivity of these responses to Wiptide, a PKA inhibitor [40]. However, the G-protein pharmacology is different in the cone cells suggesting different PKA activation pathways in DRG neurones exposed to pregabalin. There appears to be a considerable number of apparently conflicting effects mediated by PKA, although a number of different preparations have been studied. The concept of conditional protein phosphorylation such that initial phosphorylation state affects the sensitivity of different effector targets to subsequent activation [39] could determine responses to gabapentin and pregabalin acting through PKA. A future challenge will be to determine how PKA plays roles in activation and sensitization of DRG neurones and also plays a role in gabapentin and pregabalin responses to dampen down electrical excitability in the same neurones. Perhaps answers will come when we have a better understanding of the changes in neuronal phenotype that develop with pain disorders?

An interesting feature of some actions of gabapentin and pregabalin is that their effects often outlast the period of drug delivery. Maintained intracellular signalling effects of these drugs and the delayed responses involving PKA modulation of Ca^2+ ^and K^+ ^conductances may underlie this characteristic.

Gabapentin has previously been shown to attenuate high frequency action potential firing both in CNS neurones [41] and in cultured DRG neurones [5,12]. Pregabalin produces the same effect, dramatically but reversibly reducing the number of action potentials fired in response to 300 ms depolarising current commands. The underlying mechanisms involved in these reductions in electrical excitability could involve inhibition of voltage-activated Ca^2+ ^channels with resulting reduced activation of Ca^2+^-activated K+ currents and effects on voltage-dependent ion channel availability. Although the delayed enhancement of voltage-activated K^+ ^channels could contribute, this effect appears to develop over a longer period. These actions may contribute both to anticonvulsant effects of gabapentin and pregabalin as well as their therapeutic actions in pain disorders. It is particularly worth noting that the inhibition of Ca^2+ ^influx is most consistently seen in small diameter cultured DRG neurones that are likely to be the pain fibres.

Our data adds to previous work that has assessed the actions of gabapentin and pregabalin on K^+^-evoked neurotransmitter release in CNS neurones [10] and the pharmacology of pain in whole animal models [8,9,42-44]. The present work and these previous studies suggest that gabapentin and pregabalin mostly act through the same basic mechanisms. These mechanisms in cultured DRG neurones appear to involve allosteric inhibitory modulation of Ca^2+ ^channels through drug interactions with α_2_δ subunits, reduced activation of Ca^2+^-dependent conductances and modulation of both Ca^2+ ^and K^+ ^channels through metabotropic mechanisms that involve PKA. Interestingly, both gabapentin and pregabalin inhibit release of sensory peptides (substance P and CGRP) this may provide a mechanism of action down-stream of ion channel modulation. This effect is only observed after inflammation and may contribute to their analgesic properties of gabapentin and pregabalin [45].

Ca^2+ ^channels in DRG neurones are targets for antinociceptive agents but the pharmacology of these channels remains to be fully exploited in pain therapy [46,47]. Further studies on changes in ion channel subunit expression and alterations in intracellular signalling pathways in pain disorders may in the future open up new therapeutic opportunities.

## Methods

### Cell culture

One to four-day old Sprague-Dawley rats were decapitated and dorsal root ganglia removed. DRG neurones were dissociated enzymatically (0.125% collagenase for 13 minutes and 0.25% trypsin for 6 minutes) and mechanically (trituration). Primary cultures of DRG neurones were plated on lamin-polyornithine coated coverslips and bathed in Ham's F-14 culture medium (Imperial Laboratories) containing 10% horse serum (Gibco), low NGF (20 ng/ml; Sigma), NaHCO_3 _(14 mM), streptomycin (50 μg/ml) and penicillin (50 IU/ml). The cultures were maintained for up to two weeks at 37°C in humidified air with 5% CO_2_. Cultures were re-fed with fresh media after 5 days.

In some experiments DRG neurones were pre-treated with pertussis toxin (500 ng/ml; for 18 hours) to ADP-ribosylate the α subunits of certain G-proteins. This prevents pertussis toxin-sensitive G-protein being activated through a range of G-protein coupled receptors and inhibits coupling to voltage-activated Ca^2+ ^channels and other potential effectors [12,48].

### Electrophysiology and calcium imaging

The whole cell patch clamp recording method and fura-2 Ca^2+ ^imaging were used to measure the inhibitory actions of pregabalin and gabapentin on Ca^2+ ^entry through voltage-activated channels. Initially, multiple firing properties of a sub-population of DRG neurones were studied using a patch pipette solution containing in mM: KCl, 140; EGTA, 5; CaCl_2_, 0.1; MgCl2, 2.0; HEPES, 10.0; ATP, 2.0. The extracellular solution containing in mM: NaCl, 130; KCl, 3.0; CaCl_2 _2.0; MgCl_2_, 0.6; NaHCO_3 _1.0, HEPES 10.0 and glucose 5.0. For recording Ca^2+ ^channel currents the patch pipettes were filled with CsCl-based solution containing in mM: 140 CsCl, 0.1 CaCl2, 5 EGTA, 2 MgCl2, 2 ATP, 10 Hepes. The pH and osmolarity of the patch pipette solutions were corrected to 7.2 and 310–320 mOsm.l^-1 ^with Tris and sucrose. The extracellular bathing solution used to study Ca^2+ ^currents contained in mM: 130 choline chloride, 2 CaCl2, 3 KCl, 0.6 MgCl2, 1 NaHCO3, 10 HEPES, 5 glucose, 25 tetrethylammonium chloride, 0.0025 tetrodotoxin (Sigma). The pH and osmolarity of this extracellular bathing solution was corrected to 7.4 and 320 mOsml^-1 ^with NaOH and sucrose respectively. The recording solutions used in these experiments were designed to attenuate voltage-activated Na^+ ^and K^+ ^currents and isolate voltage-activated Ca^2+ ^currents. The range of values for the series resistance under our recording conditions was from ~8 to 15 MΩ. Pregabalin and other drugs were applied to the extracellular environment by low-pressure ejection from a blunt pipette positioned about 50–100 μm away from the cell being recorded. Voltage-activated Ca^2+ ^currents were evoked by 100 ms voltage step commands applied every 30 s.

Similar protocols were used to study the actions of pregabalin on voltage-activated K^+ ^currents but NaCl-based extracellular bathing solution and KCl-based patch pipette solutions were used.

In some experiments GTP-γ-S was photoreleased inside DRG neurones. Caged GTP-γ-S (100 μM; Molecular Probes) was included in the CsCl-based patch pipette solution. After obtaining control Ca^2+ ^currents intracellular flash photolysis was achieved by flashing the neurone being studied (three 200 V flashes to produce ~15μM GTP-γ-S) using a XF-10 xenon flash lamp with a UG11 bandpass filter (Hi-Tech Scientific; [49])

All voltage-activated Ca^2+ ^and K^+ ^currents had scaled linear leakage and capacitance currents subtracted to obtain values for the net inward Ca^2+ ^current or net outward K^+ ^current. Data are given as mean ± standard error of the mean (s.e.m.) values and statistical significance was determined using a paired or independent Student's *t *test as appropriate.

For Ca^2+ ^imaging in cultured DRG neurones the cultures were incubated for 1 hour in NaCl-based extracellular solution contained (in mM): NaCl, 130; KCl, 3.0; MgCl_2_, 0.6; CaCl_2_, 2.0; NaHCO_3_, 1.0; HEPES, 10.0; glucose, 5.0 and fura-2AM, 0.01; (Sigma, 1 mM stock in dimethylformamide). The pH was adjusted with NaOH to 7.4 and the osmolarity to 310–320 mOsm with sucrose. The cells were then washed for 10–20 minutes with NaCl-based extracellular solution to remove the extracellular fura-2AM and this period allowed cytoplasmic de-esterification of the Ca^2+ ^sensitive fluorescent dye. The cells were constantly perfused with NaCl-based extracellular solution (1–2 ml/min) and viewed under an inverted Olympus BX50WI microscope with a KAI-1001 S/N 5B7890-4201 Olympus camera attached. Some Ca^2+ ^imaging experiments were carried out using a Ca^2+^-free NaCl-based solution (as standard NaCl-based extracellular solution but with no added CaCl_2_) or choline chloride-based solution (as standard NaCl-based extracellular solution but with choline chloride in place of NaCl). The fluorescence ratiometric images from data obtained at excitation wavelengths of 340 nm and 380 nm were viewed and analysed using OraCal pro, Merlin morphometry temporal mode (Life Sciences resources, version 1.20). The DRG neurones were stimulated with NaCl-based extracellular solution containing high K^+ ^(30 mM), which produced depolarisation, activation of voltage-gated Ca^2+ ^channels and large transient increases in intracellular Ca^2+^. Three consistent transient increases in intracellular Ca^2+ ^could be obtained in a single experiment on cultured DRG neurones [11]. The actions of pregabalin and gabapentin (2.5–250 μM) were investigated on the response to the second stimulus in DRG neurones. The actions of pregabalin and gabapentin on the Ca^2+ ^transient amplitude, duration at 1/2 peak amplitude and total Ca^2+ ^flux were measured. All experiments were conducted at room temperature and data are expressed as means ± s.e.m.

## List of abbreviations

DRG, Dorsal root ganglion.

cAMP, Cyclic adenosine monophosphate

GABA, Gamma aminobutyric acid

GBP, Gabapentin

GTP-γ-S, Guanosine 5'-o(3-thio)triphosphate

I_Ca_, Calcium current

NGF, Nerve growth factor

PGB, Pregabalin

PKA, Protein kinase A

PP, Pre-pulse

(Rp)-cAMP, (R)-adenosine, cyclic 3', 5'-(hydrogenphosphorothioate) triethylammonium

## Authors' contributions

All the authors of this manuscript contributed to electrophysiological and Ca^2+ ^imaging experiments. DM and RHS designed and conducted the experiments on K^+ ^currents and wrote the first draft of this manuscript.
